# Variation in Population Synchrony in a Multi-Species Seabird Community: Response to Changes in Predator Abundance

**DOI:** 10.1371/journal.pone.0131543

**Published:** 2015-06-26

**Authors:** Gail S. Robertson, Mark Bolton, Paul Morrison, Pat Monaghan

**Affiliations:** 1 Institute of Biodiversity, Animal Health and Comparative Medicine, University of Glasgow, Glasgow, United Kingdom; 2 RSPB Centre for Conservation Science, Royal Society for the Protection of Birds, Sandy, Bedfordshire, United Kingdom; Institut Pluridisciplinaire Hubert Curien, FRANCE

## Abstract

Ecologically similar sympatric species, subject to typical environmental conditions, may be expected to exhibit synchronous temporal fluctuations in demographic parameters, while populations of dissimilar species might be expected to show less synchrony. Previous studies have tested for synchrony in different populations of single species, and those including data from more than one species have compared fluctuations in only one demographic parameter. We tested for synchrony in inter-annual changes in breeding population abundance and productivity among four tern species on Coquet Island, northeast England. We also examined how manipulation of one independent environmental variable (predator abundance) influenced temporal changes in ecologically similar and dissimilar tern species. Changes in breeding abundance and productivity of ecologically similar species (Arctic *Sterna paradisaea*, Common *S*. *hirundo* and Roseate Terns *S*. *dougallii*) were synchronous with one another over time, but not with a species with different foraging and breeding behaviour (Sandwich Terns *Thalasseus sandvicensis*). With respect to changes in predator abundance, there was no clear pattern. Roseate Tern abundance was negatively correlated with that of large gulls breeding on the island from 1975 to 2013, while Common Tern abundance was positively correlated with number of large gulls, and no significant correlations were found between large gull and Arctic and Sandwich Tern populations. Large gull abundance was negatively correlated with productivity of Arctic and Common Terns two years later, possibly due to predation risk after fledging, while no correlation with Roseate Tern productivity was found. The varying effect of predator abundance is most likely due to specific differences in the behaviour and ecology of even these closely-related species. Examining synchrony in multi-species assemblages improves our understanding of how whole communities react to long-term changes in the environment and suggests that changes in predator abundance may differentially affect populations of sympatric seabird species.

## Introduction

Synchronous temporal variations in demographic parameters have been documented among different populations of single species [1–5]. In contrast, synchrony among sympatric populations of different species has received much less attention [6–9]. In the limited number of studies examining synchrony in more than one species, variation in only a single demographic parameter, such as population abundance, has been analysed [6], [7], [10], [11]. Examining synchrony in larger species assemblages and incorporating several demographic parameters increases our understanding of the mechanisms responsible for influencing changes in population trends of whole communities [12], [13].

Species coexisting together in the same area are subject to the same environmental conditions. While during periods of extreme environmental change species with disparate ecological niches have been shown to exhibit synchronous population fluctuations [14], in typical conditions ecologically similar species with similar foraging and breeding behaviour might be expected to respond in the same way to changes in the environment compared with ecologically dissimilar species [15–18].

It is unclear what specific mechanisms facilitate temporal synchrony or asynchrony in demographic parameters of sympatrically breeding species. Hypotheses have suggested that shared stochastic events such as changes in weather conditions, prey availability and the abundance of generalist predators may influence population fluctuations among species [2], [3], [7], [19], [20]. Studies have shown that the presence of predators can differentially affect closely-related species with similar ecological requirements [7], [21], resulting in synchronous or asynchronous variation in population demographics [22], [23].

Breeding population abundance and productivity of seabirds have been shown to be closely correlated with food availability, weather conditions and predation pressure in breeding and wintering areas [24–28]. While various studies have examined how changes in food availability and oceanographic conditions drive temporal fluctuations in demographic parameters among sympatric species [24], [29], few have examined how variation in predator abundance influences multi-species seabird communities (but see [30], [31]). Colony-based predation can have significant deleterious effects on seabird populations [31–33], but seabird species with broadly similar ecological requirements have been shown to vary in their vulnerability to predation [31], [33].

Large gulls are opportunistic generalist predators and have been shown to negatively affect tern abundance and breeding success through direct predation of eggs and chicks as well as through competition for nest sites [31], [34], [35]. The effect of gull predation on breeding population abundance and productivity might be expected to vary among sympatrically breeding terns, as even closely-related species can show consistent interspecific differences in nesting behaviour and predator defence strategies [31], [33], [36].

In this study, we examine inter-annual variation in two demographic parameters of four tern and two large gull species breeding on Coquet Island, northeast England. We test for synchrony in long-term changes in breeding population abundance and in productivity among four tern species and compare the effect of an anthropogenically imposed decrease in large gull abundance on ecologically similar and dissimilar tern species. We aim to address two main questions in this study: 1) do ecologically similar tern species exhibit synchronous changes in demographic parameters over time and if so, is this influenced by predator abundance, and 2) do changes in predator abundance differentially affect breeding abundance and productivity of tern species.

## Materials and Methods

### Study area

Coquet Island is a small (5 ha) low-lying island, 2 km from the mainland coast of Northumberland, northeast England (55° 20’ N, 1° 32’ W). It is a Site of Special Scientific Interest (SSSI) and a Special Protected Area (SPA) and has been managed as a reserve for a seabird assemblage which includes four nationally and internationally important tern species by the Royal Society for the Protection of Birds (RSPB) since 1970. Coquet Island supports internationally important Roseate (*Sterna dougallii*) and Sandwich Tern (*Thalasseus sandvicensis*) populations (~82 and ~953 breeding pairs respectively), ~1,212 pairs of Common Terns (*S*. *hirundo*) and ~1,141 pairs of Arctic Terns (*S*. *paradisaea*) (5-year means from www.jncc.defra.gov.uk/page-4460 and www.jncc.defra.gov.uk/smp/sitesBrowser.aspx?SiteID=88850&breedingSuccessSitesOnly=FALSE). Arctic, Common and Roseate Terns have comparable body sizes (average body weights of Common Tern = 130 g, Arctic Tern = 110 g, Roseate Tern = 110 g [37]), occupy similar ecological niches and exhibit similarities in diet and foraging range. Sandwich Terns are ecologically and morphologically dissimilar to Arctic, Common and Roseate Terns [38] and exhibit very different foraging, migratory and nesting behaviour [38]. Being larger (average body weight = 250 g [37]), Sandwich Terns are able to forage more successfully during periods of adverse foraging conditions, such as in high winds, than smaller tern species and have less restrictive energy budgets [39–41].

Tern colonies were distributed homogenously across Coquet Island in the early 1970s, but after 1976 colonies became concentrated in southwestern areas following an increase in the extent of large gull territories [42]. Between 1998 and 2000, the number of breeding pairs of Herring (*Larus argentatus*) and Lesser Black-backed Gulls (*L*. *fuscus*) increased on Coquet from 11 to 49 (345%) and 20 to 184 (820%) respectively [42], [43]. The RSPB implemented a program of large gull disturbance and population control on Coquet, under licence from Natural England, annually from 2000 to present. Large gull numbers have declined to pre-1998 levels [42], [44]. Other conservation measures introduced on Coquet after 2000 include the provisioning of more nest boxes for Roseate Terns and the construction of artificial breeding areas [45].

### Demographic data collection

Demographic parameters of four tern species (Arctic, Common, Sandwich and Roseate) have been collected annually by RSPB staff on Coquet Island since 1975. Number of breeding pairs (breeding population abundance) was recorded for each species by carrying out one or two whole island nest censuses (census with the largest recorded nest count was used as the final annual breeding population abundance), from mid incubation to early chick-rearing. Overall productivity (number of fledged chicks per nest) was estimated from a subset of 30–50 Arctic and Common Tern nests from study sites located in the centre of their respective colonies, and from colony wide counts of Roseate and Sandwich Tern fledglings [46], [47].

Herring and Lesser Black-backed Gulls are the only species of large gull which regularly attempt to breed on Coquet Island and the only significant predator of resident tern species. Their numbers were quantified by a whole island census of nests each year during mid incubation (within 3 weeks of first egg being found). As nests belonging to each gull species could not be conclusively differentiated, the breeding population abundances of both species were combined for analyses. A gull control program was first implemented on Coquet Island in 2000, to try to reduce gull predation on tern species and reduce competition for nest sites through a combination of egg and nest destruction and adult disturbance [35], [42], [48]. A full description of the disturbance and egg and nest removal techniques used is provided in Booth and Morrison [42].

### Ethics statement

This study was carried out with the consent of Natural England, the advisory body responsible for granting licences for field research in England. Coquet Island is owned by the Duke of Northumberland, and the RSPB leases the island in order to carry out research and conservation work. As tern and gull species are protected under the Wildlife and Countryside Act 1981, efforts were made to ensure minimal disturbance to tern colonies during censuses, and annual gull control was consented by Natural England. The work was approved by the Coquet Island Conservation Committee which consists of a panel of researchers and conservationists charged with conserving seabird populations on Coquet and ensuring research activities achieve appropriate ethical standards.

### Data analyses

Data on the breeding population abundance and productivity of the four tern species and the abundance of the two large gull species on Coquet Island from 1975–2013 were downloaded from www.jncc.defra.gov.uk/page-4460 and www.jncc.defra.gov.uk/smp/sitesBrowser.aspx?SiteID=88850&breedingSuccessSitesOnly=FALSE. Breeding population abundance and productivity were collected annually for all four tern species from 1975 and 1991 respectively, and breeding abundance of both large gull species was recorded from 1975. The year in which productivity data collection began varied among tern species (from between 1983–1991). Consecutive annual productivity data for all four species were available from 1991, hence inter-annual changes in productivity were compared among species from 1991–2013.

A continuous time series of breeding abundance data spanning 39 years was therefore available for four tern species and two large gull species. Large gull breeding abundance data were missing for one year (2003), hence this year was removed from analyses comparing tern and gull populations. Due to difficulties in measuring productivity of Sandwich Terns on Coquet Island in some years (owing to annual variation in the position and density of the colony), productivity data for this species were severely fragmented and were therefore excluded from analyses. Productivity data for Arctic, Common and Roseate Terns were available for 23 consecutive years with no missing values. Twenty-five years of tern and large gull breeding abundance data were available before large gull control measures were implemented in 2000 after which a further 13 years of abundance data were available (excluding 2003).

Data on population size are likely to exhibit autocorrelation, and long-term trends may obscure short-term changes [49]. We tested for autocorrelation in breeding abundance data for each tern species using the ‘acf’ function from the ‘stats’ package in R (version 3.1.2). Time series exhibited significant autocorrelation if correlation coefficients for different time lags (years) were not included within 95% confidence intervals calculated using the ‘acf’ function. Significant autocorrelation (r) was found for Arctic Tern (lag 1 (r_t + 1_) = 0.83, lag 2 (r_t + 2_) = 0.73, lag 3 (r_t + 3_) = 0.63), Common Tern (r_t + 1_ = 0.74, r_t + 2_ = 0.63, r_t + 3_ = 0.50), Sandwich Tern (r_t + 1_ = 0.74) and Roseate Tern (r_t + 1_ = 0.89, r_t + 2_ = 0.79, r_t + 3_ = 0.75) breeding abundance data.

Cubic smoothing splines are commonly used to detrend time series data in ecological studies, as they have been shown to adequately represent general trends [50–53]. Various smoothing splines with different numbers of knots and degrees of freedom were considered, and the spline which best represented changes in breeding abundance for all four tern species was selected. Long-term trends were removed from time series by subtracting actual data values from the smoothing spline (fitted using the smooth.spline function available in the ‘stats’ package in R), where the number of knots in the spline was equal to the number of decades in the time series (rounded) + 1 [50], [54], [55]. The resulting values were then standardised by dividing each by the standard deviation of the detrended time series. This was repeated for each tern species.

To address the first question outlined in the aims (do ecologically similar tern species exhibit synchronous changes in demographic parameters over time and is this influenced by predator abundance), mean cross-correlation coefficients and associated bootstrapped confidence intervals were calculated (using the ‘mSynch’ function from the ‘ncf’ package in R [20]) among all four tern species using breeding population abundance and productivity data. Mean cross-correlation coefficients were then calculated to examine changes in breeding abundance and productivity among only ecologically similar species (Arctic, Common and Roseate Terns). To examine synchrony in inter-annual changes in demographic parameters, detrended and standardised breeding abundance data were used in correlations, after testing data for normality using Shapiro-Wilks tests. This analysis was repeated using raw data values for breeding abundance, to test for synchrony among population trends. Annual productivity data were not detrended, as time series did not exhibit autocorrelation.

To examine whether gull abundance causes synchrony in inter-annual changes in population demographics among tern species, mean cross-correlations with bootstrapped confidence intervals were calculated using detrended and standardised breeding abundance data and raw productivity data before (1975–1999) and after (2000–2013) gull control was implemented in 2000.

To address the second question outlined in the aims (do changes in predator abundance differentially affect breeding abundance and productivity of tern species), we used cross-correlation functions (carried out using the ‘ccf’ function in R) [1–3], [55]. Cross-correlation functions were used to calculate Pearson’s product-moment correlation coefficients between large gull and tern abundance at different time lags using data from 1975–2013. Breeding abundance data used in cross-correlations were detrended and standardised to remove the trend from the data and to ensure values were comparable among species. As disturbance and predation by large gulls in one year may influence the number of terns returning to breed on the island in subsequent years, we examined correlations between large gull abundance at lag 0 and tern abundance at lags 1–3 (juvenile terns which fledged on Coquet usually returned to breed within 2–3 years [56]). Species with cross-correlation coefficients that were not included within 95% confidence intervals were regarded as significantly correlated. This analysis was repeated to test for correlations between tern productivity and large gull abundance at each time lag. Productivity data were not detrended, as autocorrelation was not evident in productivity time series. Analyses were carried out in R version 3.1.2 [57].

## Results

### Temporal synchrony in tern demographic parameters

**Fig 1:**
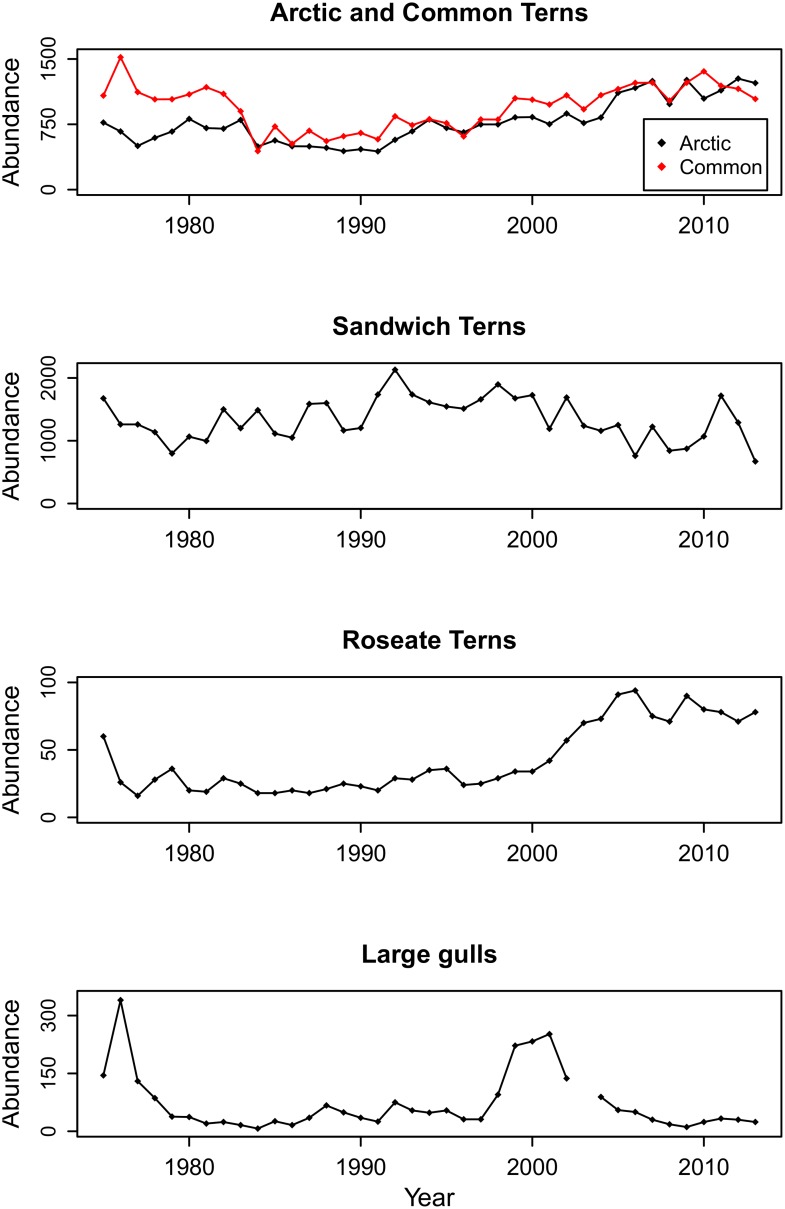
Breeding population abundance of four tern species and two large gull species on Coquet Island. Time series showing Arctic Tern (black), Common Tern (red), Sandwich Tern, Roseate Tern and large gull (Herring and Lesser Black-backed) breeding population abundance (number of breeding pairs) on Coquet Island from 1975–2013. One year (2003) of large gull breeding population abundance is missing.

Arctic, Common and Roseate Terns showed similar overall trends in breeding population abundance from 1975–2013. All three species exhibited declines in breeding abundance in the mid 1980s, but abundance increased again after 2000 (Fig 1). Sandwich Terns did not show any clear trend in breeding abundance over time due to large fluctuations in annual populations, but the number of breeding pairs on Coquet appeared to decrease after 2000 (Fig 1).

Mean cross-correlation coefficients and bootstrapped confidence intervals were calculated using detrended breeding abundance data and raw productivity data for all four tern species. When examining inter-annual changes in breeding abundance using detrended data, the mean cross-correlation coefficient was relatively low for Arctic, Common, Sandwich and Roseate Terns (r = 0.14, 95% confidence intervals = -0.20, 0.39, replicates = 1000), and bootstrapped confidence intervals overlapped zero. However, when Sandwich Terns were excluded from this analysis the mean cross-correlation coefficient was higher and bootstrapped confidence intervals did not overlap zero (r = 0.26, 95% confidence intervals = 0.03, 0.39). Examining similarity in trends of breeding abundance (using raw data values) showed that correlation among all four tern species was also found to be significantly lower (r = 0.19, 95% confidence intervals = -0.36, 0.87) than when Sandwich Terns were excluded (r = 0.71, 95% confidence intervals = 0.59, 0.87).

This analysis was repeated for 1975–1999 and 2000–2013. No synchrony was apparent in inter-annual changes in breeding abundance among all four tern species before or after the introduction of large gull control. Although we found no significant correlation among Arctic, Common and Roseate Terns prior to 2000, there was a significant correlation among these three species after gull control was introduced in 2000 (Table 1).

**Table 1:** Results of mean cross-correlation tests calculated from detrended and standardised breeding population abundance data and raw productivity data, examining correlations in inter-annual changes among tern species before (1975–1999) and after (2000–2013) gull control was implemented.

	1975–1999	2000–2013
**Breeding population abundance**		
Arctic, Common,	r = 0.03, 2.5% = -0.21,	r = 0.26, 2.5% = -0.25,
Sandwich, Roseate	97.5% = 0.46	97.5% = 0.49
Arctic, Common, Roseate	r = 0.20, 2.5% = -0.19,	r = 0.47, 2.5% = 0.46,
	97.5% = 0.46	97.5% = 0.49
**Productivity**		
Arctic, Common, Roseate	r = 0.52, 2.5% = 0.30,	r = 0.62, 2.5% = 0.49,
	97.5% = 0.78	97.5% = 0.74

**Fig 2:**
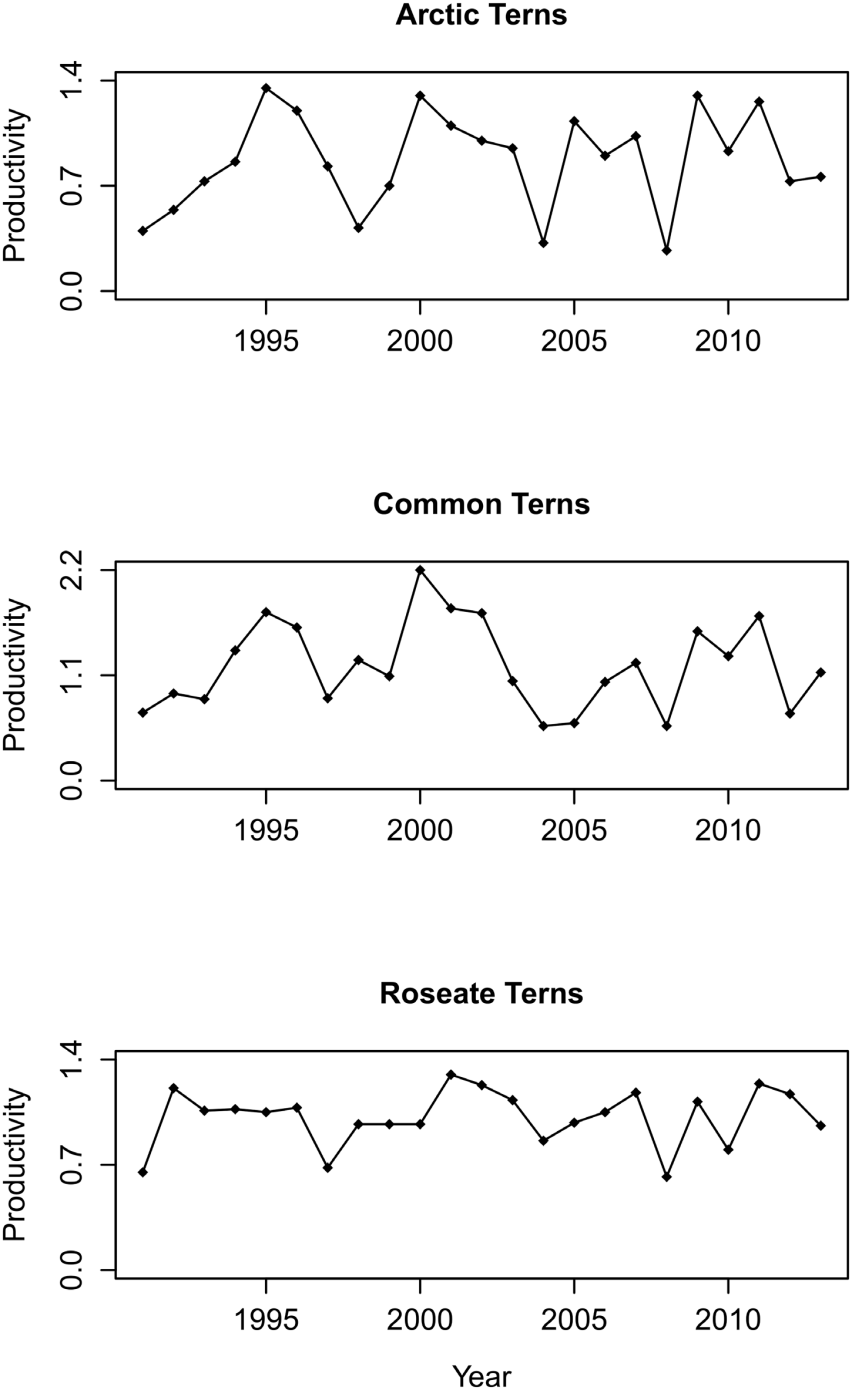
Productivity of three tern species on Coquet Island. Time series showing annual productivity (number of fledged chicks per nest) of Arctic, Common and Roseate Tern populations breeding on Coquet Island from 1991–2013.

Inter-annual changes in productivity appeared to be similar for Arctic, Common and Roseate Terns (Fig 2). A mean cross-correlation coefficient calculated for all three species using raw data values was found to be significant (r = 0.58, 95% confidence intervals = 0.49, 0.74), suggesting that changes in productivity were synchronous among Arctic, Common and Roseate Terns. Productivity of Sandwich Terns was not recorded consistently on Coquet hence no comparisons could be made with productivities of other tern species.

### Changes in large gull abundance and tern demographic parameters

To examine how predator abundance influences inter-annual changes in population demographics of the tern species, large gull abundance data from 1975–2013 (excluding 2003) were compared with the breeding population abundance of each tern species. Fig 1 appears to show that Arctic, Common and Roseate Tern breeding abundance increased following a decline in large gull breeding abundance after the commencement of a gull control program in 2000.

**Fig 3:**
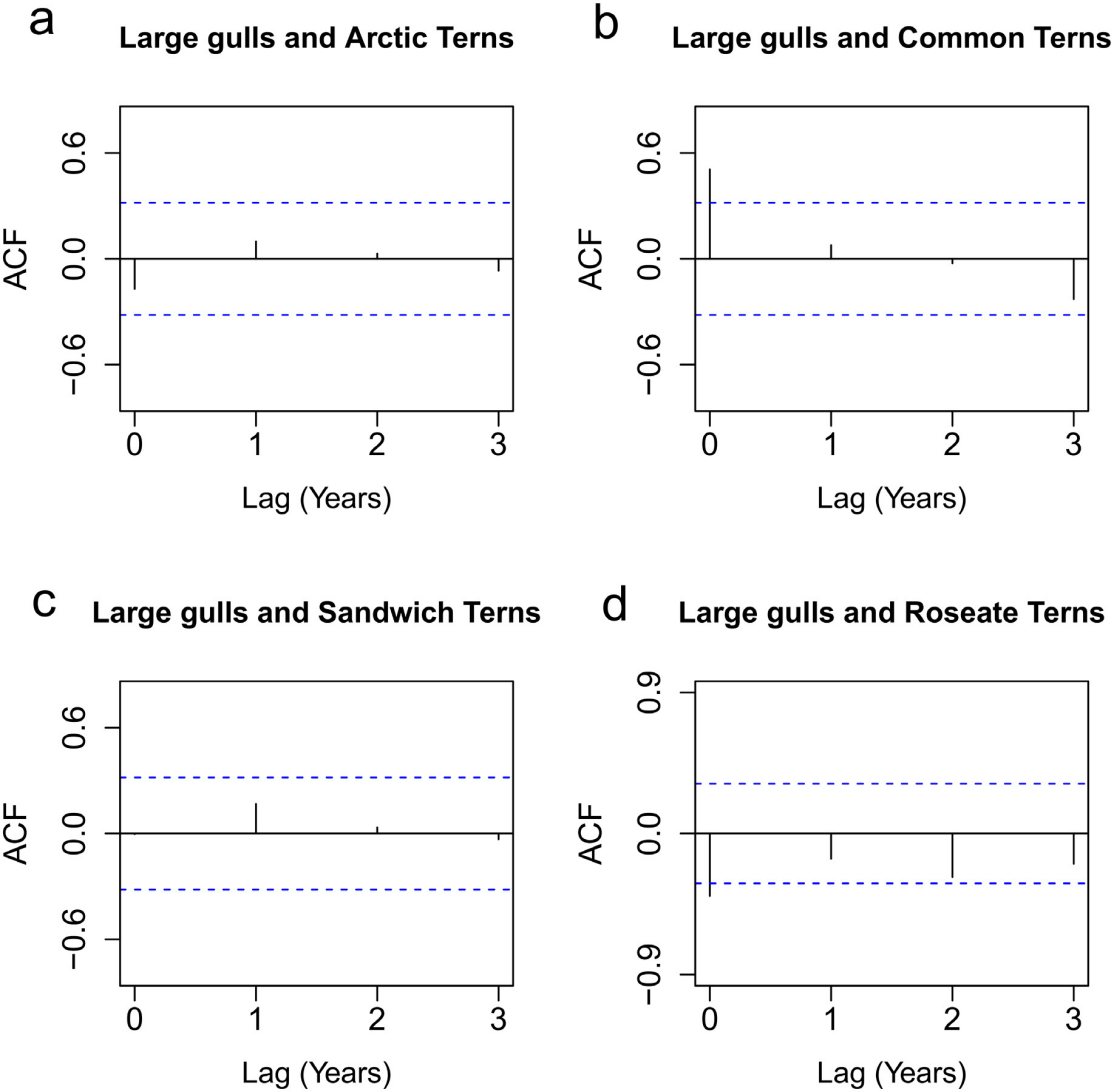
Cross-correlation functions calculated for large gull and tern detrended and standardised breeding population abundance (number of breeding pairs) from 1975–2013 (excluding 2003) for a) large gull and Arctic Terns, b) large gull and Common Terns, c) large gulls and Sandwich Terns and d) large gulls and Roseate Terns. A significant negative correlation (where the correlation coefficient lay outside the lower 2.5% confidence interval) was found at lag 0 for Roseate Terns (r_t_ = -0.40). There was a significant positive correlation between large gull and Common Tern abundance at lag 0 (r_t_ = 0.51). There were no significant correlations between large gull and Sandwich Tern and large gull and Arctic Tern breeding population abundances at any time lag.

Cross-correlation coefficients were calculated to compare detrended and standardised breeding abundance of large gulls with those of the four tern species breeding on Coquet for 3 positive time lags (years) from 1975–2013 (Fig 3). No significant correlations were found between large gull and Arctic Tern breeding population abundance or between large gull and Sandwich Tern breeding population abundance at any time lag (Fig 3a and 3c). Conversely, there was a significant positive correlation between abundance of large gulls and Common Terns at lag 0 (r_t_ = 0.51; Fig 3b). Fig 3d shows that the breeding abundance of large gulls and that of Roseate Terns were significantly negatively correlated during concurrent years (r_t_ = -0.40).

**Fig 4:**
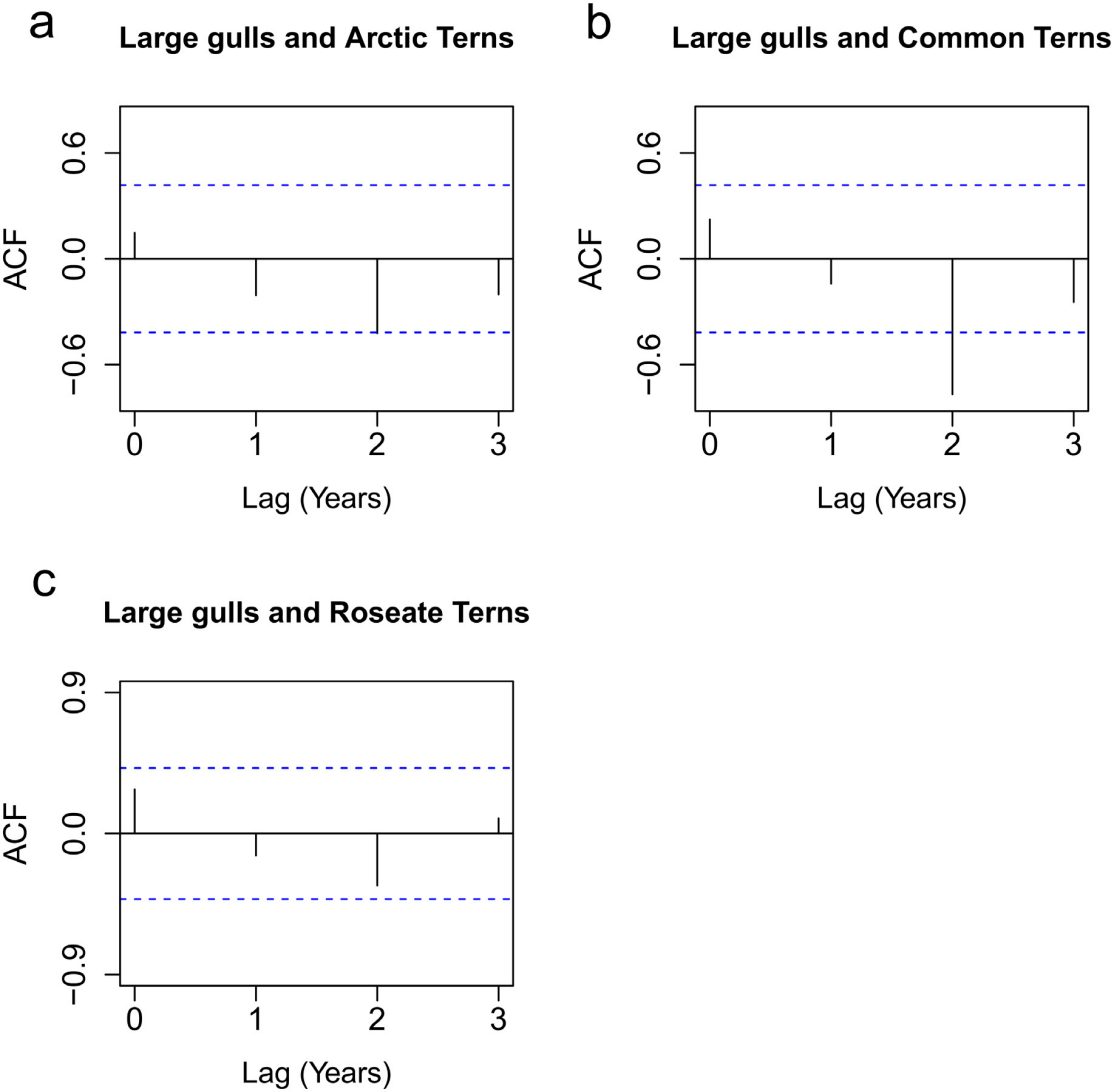
Cross-correlation functions calculated for large gull detrended and standardised breeding population abundance (number of breeding pairs) and tern productivity data from 1991–2013 (excluding 2003) for a) large gull and Arctic Terns, b) large gull and Common Terns and c) large gulls and Roseate Terns. A significant negative correlation (where the correlation coefficient lay outside the lower 2.5% confidence interval) was found at lag 2 for Arctic (r_t+2_ = -0.51) and Common Terns (r_t+2_ = -0.77). There was no significant correlation between large gull breeding abundance and Roseate Tern productivity at any time lag.

Cross-correlation coefficients were also used to compare how large gull breeding abundance influenced productivity of Arctic, Common and Roseate Terns at different time lags (Fig 4). Fig 4a and 4b show that detrended and standardised large gull breeding abundance and productivity of Arctic and Common Terns were significantly negatively correlated at lag 2 (r_t+2_) years (r_t+2_ = -0.51 and -0.77 respectively). This suggests that annual increases in large gull abundance could negatively influence Arctic and Common Tern productivity two years later. Gull abundance did not influence Roseate Tern productivity at any time lag (Fig 4c).

## Discussion

Our results show that changes in annual breeding population abundances of Arctic, Common and Roseate Terns on Coquet Island were synchronous between 1975 and 2013, but there was no synchrony in breeding abundances of Arctic, Common, Roseate and Sandwich Terns. This suggests that temporal variations in Sandwich Tern breeding abundance were not synchronous with those of other tern species breeding on Coquet. Temporal changes in annual productivity were also synchronous among Arctic, Common and Roseate Terns.

Synchrony in changes in productivity among species may be explained by general similarities in chick provisioning behaviour, such as diet and foraging range [38]. Productivity is likely to be influenced by local conditions during the breeding season which affects the ability of parents of all three species to deliver food to chicks, whereas the number of pairs that return annually to nest on the island may be affected by various factors such as conditions in wintering grounds and on migration, and availability of suitable nesting habitat. As UK tern species vary in wintering habitat and migration strategies [38], as well as in preference for nesting areas, interspecific synchrony in breeding population abundance is less likely than synchrony in productivity.

Changes in abundance among all four tern species were not significantly similar before or after the introduction of gull control, whereas changes were only significantly similar among Arctic, Common and Roseate Terns after 1999, when gull numbers were controlled. Inter-annual changes in productivity were significantly similar among three tern species before and after gull abundance decreased. Hence, similarities in changes in population demographics do not appear to have been influenced by the number of large gulls breeding on Coquet. This may be expected given the varying effect large gull abundance had on different tern species. However, it should be emphasised that as there were only a limited number of years where the number of breeding gulls was particularly high, we may have been unable to detect a synchronisation effect in this study.

Our study suggests that factors other than predator abundance may influence synchronisation in changes in demographic parameters. Studies have shown that sympatrically breeding seabird species can exhibit similar fluctuations in population demographics in response to general changes in the marine environment [8], [12]. While diet and foraging behaviour of Arctic, Common and Roseate Terns breeding at various colonies including Coquet Island have been shown to vary [58–60], there are some general similarities in prey preferences and nesting behaviour among these species which may influence changes in demographic parameters [15], [38].

Tern species have been identified as being especially vulnerable to reductions in sandeel abundance, due to their relatively short foraging ranges, restricted dietary preferences and limited diving ability [39]. However, it has been suggested that Sandwich Terns are less at risk from food shortages than Arctic, Common and Roseate Terns due to their longer foraging ranges, wider diets and ability to exploit prey deeper in the water column [38], [39], [61].

While synchrony in breeding population abundance and productivity was evident among Arctic, Common, and Roseate Tern populations on Coquet, dissimilarities between these species and Sandwich Tern breeding population abundance suggest that Sandwich Terns responded differently to changes in the shared environment. However, previous studies have reported that large scale environmental changes can synchronise population fluctuations of different vertebrate species regardless of ecological niche [14], hence populations of all four tern species may fluctuate in synchrony during extreme conditions.

Our results suggest that changes in the abundance of generalist predators can differentially affect population demographics of sympatrically breeding seabird species. The breeding population abundance of Roseate Terns was found to increase significantly following corresponding declines in large gull abundance, while the breeding population abundance of Common Terns was found to be positively correlated with that of large gulls. Arctic and Sandwich Tern populations did not respond to changes in large gull abundance. This may be due to reduced vulnerability of Sandwich Terns to predation owing to their high colony densities and reluctance to flush from nests [62], or due to differences in diet and foraging behaviour of these species [38], [63]. Arctic and Common Terns have effective predator defence strategies and are highly aggressive during the breeding season [38]. These species tend to have wider diets than Roseate Terns and often forage in similar areas [58], [64]. Roseate Terns may be more vulnerable to disturbance from gulls than other tern species as they arrive at breeding sites comparably late in the breeding season, are less aggressive than Arctic and Common Terns, and lack nest defence strategies as effective as those of other tern species [38]. Roseate Terns naturally nest under boulders and in rocky crevices and are easily disturbed by the activities of predators, hence the provisioning of nest boxes and predator control is most likely to benefit this species [45]. It is unclear why Common Tern populations were positively affected by increases in large gull abundance. Common Terns are highly aggressive during chick-rearing [65], making this species less at risk from disturbance and predation. Perhaps Common Terns benefited from gulls predating and disturbing Roseate Terns, although exactly how predation on one species benefits another is not clear.

Arctic and Common Tern productivity were negatively correlated with increases in large gull populations, while gull abundance did not appear to affect Roseate Tern productivity. Nest boxes are provided for Roseate Terns on Coquet which is likely to reduce gull predation on eggs and chicks [45], perhaps explaining why gull abundance was not found to influence productivity of this species. Fledgling Roseate Terns are also able to shelter from predators in boxes and among boulders surrounding boxes. Productivity of Arctic and Common Terns were found to be negatively correlated with large gull abundance two years previously. As Arctic and Common Terns breed for the first time at 2–3 years old [56], increases in gull abundance in year of hatching may influence the productivity of tern fledglings when they return to breed. However, these results should be interpreted with caution, as large gull abundance was only very high (over 150 pairs) on Coquet in some years, hence the effect of disturbance and predation may be difficult to detect in some tern species.

The abundance of Roseate Terns breeding on Coquet exhibited a general increase after 1999. However, this population increase cannot fully be attributed to the introduction of gull control, as abundance could have increased for other reasons, such as the introduction of nest boxes in the 1990s, an increase in the number of nest boxes provided in 2000 and general improvement of breeding habitat on the island [42], [45]. Hence, the significant negative cross-correlation between the abundance of large gulls and Roseate Terns from 1975–2013 does not provide conclusive evidence that anthropogenic declines in large gull abundance influence tern populations. However, our results suggest that the control program on Coquet Island may at least partly influence changes in endangered Roseate Tern populations, but long-term tern demographic data from a nearby colony where no large gull control had been implemented is necessary to confirm or refute the results of this study.

Measuring synchrony in demographic parameters at a multi-species seabird assemblage improves our understanding of how whole communities react to long-term variations in the marine environment. Such studies are invaluable when considering the effect of climate change and other long-term environmental changes on communities of apex predators [66–68]. Our results suggest that predator abundance may be partly responsible for the interspecific variation in temporal fluctuations of tern population demographics observed on Coquet Island, but further evidence is required before the effect of gull predation can be fully understood.
